# Investigation of chronic limb ulcers in Northern Cameroon: a socio-anthropological and clinical perspective

**DOI:** 10.1186/s12879-025-11251-4

**Published:** 2025-07-01

**Authors:** Theodore Alex Tonye, Armelle Viviane Ngomba, Linda Esso, Chanceline Bilounga, Nadia Mandeng, Ingrid Kenko, Patricia Mendjime, Telesphore Guiswe, Loic Kongne Choupo, Sebastien Douka, Armand Zra, Hoche BlackBoye, Grâce Anita Nkoro, Hamza Gaya, Etienne Guenou, Donald Adegono, Leonel Keptchuime, Hermann Landry Munshili Njifon, Chevalier Nyat, Ronald Perraut, Zakari Yaou Alhadji, Georges Alain Etoundi Mballa

**Affiliations:** 1 Department for Disease, Epidemics and Pandemics Control, Yaounde, Cameroon; 2 University of Douala, Douala, Cameroon; 3 University of Yaounde 1, Yaounde, Cameroon; 4 Public Health Emergency Operation Center, Yaounde, Cameroon; 5 University of Bamenda, Bamenda, Cameroon; 6 Cameroon Field Epidemiology Training Program (CAFETP), Yaounde, Cameroon; 7 North Regional Delegation of Public Health, Garoua, Cameroon; 8 Garoua Regional Hospital, Garoua, Cameroon; 9 National Public Health Laboratory, Yaounde, Cameroon; 10 Centre Pasteur of Cameroun, Annex of Garoua, Garoua, Cameroon; 11 University of Garoua, Garoua, Cameroon

**Keywords:** Chronic limb ulcers, Neglected tropical diseases, Dermohypodermatitis, Buruli ulcer, Cameroon

## Abstract

**Background:**

In September 2023, fifty cases of chronic limb ulcers of unknown origin were reported in six Health Districts (HDs) in Northern Cameroon. This disease, locally called “Ladde”, was described as of mystical origin, transmitted by insect bites. We aimed to describe the cases, identify the cause and socio-anthropological considerations.

**Methodology:**

We conducted a mixed descriptive cross-sectional study in November 2023. A case was any person that had presented a skin ulcer on any of the four limbs for at least 4 weeks any time during the study period, suspected of infectious cause or contamination, associated or not to other conditions and residing in the study area from January 2018 to October 2023. After active case-finding in health facilities and within the community, we featured sociodemographic (sex, age, occupation), clinical (location, signs/symptoms, ulcer occurrence), and therapeutic data (itinerary, treatment and outcome). We collected blood samples, ulcer swabs and skin biopsies to test for pathogens (*Haemophilus ducreyi, Treponema pallidum, Mycobacterium ulcerans*, *Mycobacterium leprae*, *Leishmania*), performed an entomological survey to search for potential vectors and conducted a socio-anthropological survey (individual interviews and focus group discussions) to explore community perceptions.

**Results:**

We identified 153 cases in total: 119 (77.8%) were men. The median age was 38.5 years (9 months to 94 years). Farmers (*n* = 63, 41.2%), followed by housewives (*n* = 24, 15.7%) were the most affected. The lower limbs (*n* = 138, 90.2%) were the preferred location. Pain (*n* = 130, 85.0%), swelling (*n* = 113, 73.9%), ulceration (*n* = 43, 28.1%) and fever (*n* = 42, 27.5%) were the most frequent signs/symptoms at the beginning. In 79 (51.6%) cases, the ulcers occurred spontaneously and 67 (43.8%) after trauma (road injuries, blunt objects ulcers). For treatment, 129 (84.3%) cases visited a traditional healer who ordered decoctions (*n* = 98, 64.1%) and poultices (*n* = 95, 62.1%) using powder; 81 (52.9%) cases visited a health facility and received Cloxacillin (*n* = 78, 51%) and diclofenac (*n* = 70, 45.8%). Ten (6.5%) cases were completely cured. Six out of ninety-four (6.4%) cases tested were HIV positive, 8 (8.5%) were syphilis positive, all referred for appropriate care. Dermohypodermatitis (*n* = 14 out of 28, 50%) and pyogenic granuloma (*n* = 12 out of 28, 43%) were the main anatomopathological findings. No patient was positive for *Mycobacterium ulcerans, Haemophilus ducreyi or Treponema pallidum pertenue.* The entomological investigation did not reveal any potential insect vectors for leishmaniasis. Socio-anthropological survey mostly reported that “Ladde” is a disease of diabolic origin caused by a spirit which comes from a demon-possessed animal or tree.

**Conclusion:**

Posttraumatic leg ulcers and dermohypodermatitis were the predominant clinical and anatomopathological patterns. Traditional practitioners were the main point of care. Strengthening the capacity of health and laboratory personnel in the diagnosis and management of chronic skin ulcers pathogens is recommended to improve the outcome of chronic ulcers.

**Supplementary Information:**

The online version contains supplementary material available at 10.1186/s12879-025-11251-4.

## Introduction

Chronic skin ulcers are characterized by persistent skin loss (more than six weeks) that fails to heal spontaneously (after three months or more), often leading to significant morbidity [1]. It is a relatively common condition in adults, with repercussions for the patient's daily life, including pain and depression, and reduced quality of life [1]. Causes of chronic skin ulcers can be infectious and non-infectious. Common systemic causes for chronic skin ulcers include venous disease, arterial disease and neuropathy. Less common causes are metabolic disorders, haematological disorders and infectious diseases [1]. Skin ulcers of infectious origin are defined as a skin loss with no spontaneous tendency to heal due to bacterial, parasitic, fungal or viral causes. Known causes include bacterial entry into or parasitic infestation of skin tissue in tropical climates [1]. However, there is still much that is not understood about the aetiology of some endemic ulcers. The infectious causes of chronic skin ulcers should be investigated when individuals return from temporary stays in tropical regions or live in areas of known endemic tropical skin diseases. Bacterial causes include leprosy (*Mycobacterium leprae*), Buruli ulcer (*Mycobacterium ulcerans*), *Haemophilus ducreyi,* tropical phagedenic ulcer (*Bacillus fusiformis*), yaws (*Treponema pallidum* spp. *pertenue*), syphilis (*Treponema pallidum* spp. *pallidum*) and polymicrobial infections (*Streptococcus pyogenes*, *Staphylococcus aureus* spp., *Pseudomonas aeruginosa*) [2–5]. *Streptococcus pyogenes* (group A, beta-hemolytic) is a gram-positive bacterium responsible for a range of skin infections but becomes severe when it invades the bloodstream [6]. Bacterial infectious ulcers can be complicated by infection of deeper structures, such as the hypodermis, which presents clinically as erysipelas and shows large inflamed areas. Fascial compromise (fasciitis) can lead to necrosis, requiring surgical treatment in addition to antibiotics and being fatal in approximately 30% of cases [6].

The main parasitic cause is leishmaniasis (more than 20 spp. of the etiological agent; *Leishmania*), which is transmitted by the bite of an infected phlebotomine fly. The parasite causes highly incapacitating cutaneous, mucocutaneous, and visceral disease (most severe form) [7]. Visceral leishmaniasis where the parasite migrates to internal organs, is fatal if left untreated [8]. Cutaneous leishmaniasis is diagnosed using clinical examination combined with parasitological, serological (TDR) or histological tests [9, 10].

Several diagnostic methods for infectious causes exist, ranging from simple clinical examinations to complex methods. In the case of Buruli ulcer, the first clinical signs are painless nodules and swellings, usually on the arms and legs and sometimes on other parts of the body. These areas can then ulcerate and the large ulcers formed have a white and yellow base. In cases of doubt, the World Health Organization (WHO) has identified four standard laboratory methods for confirming Buruli ulcer [11]. These methods include polymerase chain reaction (PCR), direct microscopic examination, histopathological examination and culture. Bacterial dermohypodermatitis presents clinically as a large, acute red leg patches. Bacteriological tests, such as Gram staining and culture, are recommended prior to antibiotic therapy. Preferable samples are those obtained by closed blister punctures, subcutaneous punctures, biopsy, or viable fragments over swabbing the usually contaminated open lesions. Blood cultures are sometimes positive. Debridement of necrotic lesions is recommended.

The greatest burden of disease from chronic ulcers of infectious origin is found in West and Central Africa, where the highest numbers of cases are reported in Côte d'Ivoire, Ghana, Cameroon, and the Democratic Republic of the Congo (DRC) [12]. These countries share a tropical climate and dense vegetation (forest, savannah). The estimated incidence rates include 21.5 per 100,000/year in some regions of Benin and 20.7 per 100,000/year in Ghana as a whole, with rates of up to 158.8 per 100,000/year in some affected districts [12].

Cameroon has high transmission risk for neglected tropical diseases, including chronic limb ulcers of infectious origin. On 28th September 2023, the Cameroon Department for Disease, Epidemics and Pandemics Control was notified of a cluster of 50 cases of chronic limb ulcers of unknown origin in 6/15 Health Districts (HDs) of the Northern Region (Bibemi, Garoua 1, Gaschiga, Guider, Lagdo, and Pitoa). Seven similar cases observed at Gaschiga HD were previously investigated in January 2020 by a joint team from the Northern Regional Delegation for Public Health and the affected HD [13]. These were classified as post-traumatic lesions (43%), papules after insect bites (43%) and pustular lesions (14%). Laboratory analysis (pus examination) isolated *Streptococcus pyogenes, Staphylococcus aureus, Klebsiella pneumoniae,* and *Escherichia coli* in the samples [13]. Blood cultures were all negative. Buruli ulcer was clinically suspected but *Mycobacterium ulcerans* was not isolated. Locally, this condition is known as “Ladde”. The community's perceptions of “Ladde” was that of a mystical disease that cannot be cured with a conventional medical approach. Cases are more likely to be treated by traditional healers.

Given the resurgence of some 50 new cases of “Ladde” in the Northern Region, a multidisciplinary team was deployed on-site in collaboration with the different healthcare providers to trace cases in the study area, to analyse the eventual infectious causes of the ulcers in the study population, the demographic, epidemiological and clinical characteristics, the entomological environment related to the transmission of leishmaniasis, and the perceptions of the condition and treatment practices by the community and health providers.

## Methodology

### Study area

The Northern Region (one of the ten regions in Cameroon) covers an area of 66,477 km2 and is home to more than 3,197,146 inhabitants [14]. The climate is tropical savanna, with high temperatures and annual rainfall ranging from 1,200 to 900 mm, which decreases with latitude from south to north [15]. The six-month cold dry season from November to February is followed by increasingly hot months until the rains arrive. These interannual irregularities characterize the climate and give rhythm to the people’s occupations, mainly farmers, cattle breeders and a few Peul-Bororo nomads. Agriculture and livestock farming account for 90% of the population’s livelihoods [15]. Fishing increased sharply with the creation of the Lagdo hydroelectric dam and reservoir. The region is subdivided into 15 HDs (9 urban HDs and 6 rural HDs) for 150 health areas. There are 265 public health facilities and 46 private health facilities. The study area was the 6 HDs of the Cameroon Northern region who notified more than five cases to the Cameroon Department for Disease, Epidemics and Pandemics Control on September 2023. These were five urban HDs: Garoua 1, Gaschiga, Lagdo, Ngong and Pitoa, and one rural HD: Bibemi (Fig. 2). Three HDs which reported fewer than five cases (Guider, Poli, Tchollire), and six HDs which reported no cases (Garoua 2, Figuil, Golombe, Mayo-Oulo, Poli, Rey-Bouba, Touboro) were not included in the study.

### Types of study

This was a mixed descriptive cross-sectional study with quantitative and qualitative parts. The quantitative part included epidemiological, clinical, biological, therapeutic and entomological aspects. The case definition was any person that had presented a skin ulcer on any of the four limbs for at least 4 weeks any time during the study period, suspected of infectious cause or contamination, associated or not to other conditions and residing in the study area from January 2018 to October 2023. The qualitative part mainly concerned the socio-anthropological survey including any person aged 18 and over residing in the study area and persons meeting one of these criteria: any person meeting the case definition, any health personnel, traditional practitioner or other caregiver practicing in the study area. All participants provided consent.

### Quantitative part of the study

#### Epidemiological, clinical and therapeutic details

Active case detection was conducted. Health facilities (reception, emergency and hospitalization departments) and other care structures (traditional healers, traditional birth attendants) registers from January 2018 to October 2023 were reviewed to find cases that had not been reported. Suspected cases contacts were also evaluated at community level. The variables collected were: Sociodemographic characteristics (age, sex, occupation, level of education, ethnicity, marital status, urban/rural status, number of people in the household, history of travel before/after the disease) and clinical data (circumstances of occurrence, first signs, evolution, clinical description of ulcers, health care provider, and therapeutic measures). Clinical examinations were performed by a dermatologist or general practitioner.

#### Biological examinations: Identification of etiological agent(s)

For each ulcer, a laboratory biologist collected three swabs of the lesions for bacteriological analysis (*Haemophilus ducreyi*), and molecular analysis (PCR) for yaws, leishmaniasis, leprosy, Buruli ulcer and atypical mycobacteria; a trained and qualified biologist or phlebotomist collected two whole blood samples for serological analysis, including Human Immunodeficiency Virus (HIV) serology, Treponema Pallidum Haemagglutinations Assay/Venereal Disease Research Laboratory (TPHA/VDRL); and a dermatologist collected one skin biopsy for anatomopathological analysis.

Samples were stored in a cool box containing ice packs, and transported to the laboratory of the Centre Pasteur du Cameroun Annexe de Garoua (CPCAG) within six hours of collection. Serological analyses were performed at Centre Pasteur du Cameroun Annexe de Garoua (CPCAG), where aliquots were prepared and some of the samples (swabs and biopsies) transferred to the CPCY. Bacteriological, molecular, and anatomopathological analyses of skin biopsies were performed at Centre Pasteur du Cameroun Yaounde (CPCY).

#### Entomological survey

The entomological survey was carried out during the investigation by two entomologists from the National Veterinary Laboratory. To identify potential vector species at different sites, they used household spraying and larval collection techniques. Household spraying was first carried out in the homes of cases, as well as in some randomly selected houses in the neighbourhood. Larval collection consisted of a systematic search of artificial *Phlebotomus spp.* breeding sites (old canaries, containers, and tins) around the houses in the vicinity of the households. Larvae from stages L1 to L3 and L4 were collected, packaged, and brought back to the laboratory. These larvae were treated and allowed to emerge. Adults were collected via CDC-type light traps with carbon dioxide (CO2) baited from 6 p.m. to dawn. Georeferenced biconical traps were installed in various investigation localities to capture *Tabanidae chrysops* flies.

### Qualitative part of the study: Socio-anthropological survey

We conducted a survey via individual interviews and focus group discussions (FGDs) using an interview guide developed for this study (uploaded as supplementary file).

#### Individual interview survey

Semi-structured individual interviews were organized in three health districts (Garoua 1, Gaschiga and Pitoa) selected by convenience. In each of the selected districts, five people were interviewed (two cases, one community leader, one healthcare staff member and one traditional practitioner). An interview grid was used as a guide, and the interviews were recorded via smartphones. The turn to speak was respected. A dedicated space was set aside to ensure confidentiality. Each interview lasted between 30 and 45 min. The main variables were the description of ulcers, knowledge of causes, manifestations of Ladde, treatment protocol, social considerations and therapeutic itinerary.

#### Focus group discussion survey

In line with the FGD methodology, the size of each group was set at 10 homogeneous participants, with one group each in three districts selected by convenience (Bibemi, Ngong and Lagdo). Group discussions were conducted via a predesigned discussion guide. The main aspects of the discussion were the knowledge on Ladde (causes, manifestations), social beliefs and therapeutic itinerary. All discussions took place in an appropriate space that also guaranteed the confidentiality of all participants (community hut or a room within the HD). If necessary, a local translator ensured that the questions asked by the investigators, trained in the best practices and techniques of qualitative data collection, were faithfully transmitted. Time management was of the utmost importance to meet deadlines, and the intervention of all participants on questions was respected to ensure that all opinions were heard. All group discussions were recorded on smartphones with the consent of the interviewees. Field notes and observations were taken by the investigators to support the audio recording.

### Data collection and analysis

#### Quantitative data

All data were entered into Epi Info Version 7 software and an Excel 2016 spreadsheet. The sociodemographic characteristics of the participants are presented in a table as frequencies and proportions (%). Using a map, we represented the distribution of cases by HD and by Health Areas (HA) of origin. Information on where cases sought care was used to determine the therapeutic itinerary. Laboratory results and entomological findings were analysed by the biologists and entomologists; we presented these results as stated in their reports. Descriptive results are presented in the form of tables, maps, and figures.

#### Qualitative data

Following field data collection, audio transcriptions were typed into Microsoft Word software. These documents were then analysed via NVivo software. The data were then grouped by items: 1) social beliefs on the disease Ladde; 2) manifestations of Ladde; 3) causes of Ladde; 4) treatment of Ladde; and 5) prevention of Ladde. The content analysis of the qualitative data aimed to provide a dynamic clarification of meaning. Socio-anthropological data were cross-referenced with clinical findings to better understand the condition and the control measures.

### Administrative authorizations and ethical considerations

Administrative authorizations from the Ministry of Public Health and the Northern Regional Delegation for Public Health were obtained. All activities were carried out in strict compliance with administrative requirements and involved stakeholders at all health system levels, notably Central, Regional, District, health facilities and, finally, the community. The study qualified for exemption from ethical review as it was conducted as part of an outbreak investigation according to the Article 25 of the “LAW N° 2022/008 of 27 April 2022 relating to medical research involving human beings in Cameroon” [16]. The statement is: “A research project may only be carried out in an emergency situation if the risks and constraints inherent in the project are minimal and the project provides the opportunity to expect essential results that could provide long-term benefit to people suffering from the same disease or disorder, or whose state of health is comparable.” The study adhered to the Declaration of Helsinki. The participant written consent was mandatory for all the participants before interview, taking pictures and sample collection. Informed consent was obtained after a clear explanation of the study, the whole procedure and the benefits. Participants’ cultural sensitivities were respected. Participants'identities were coded by assigning a unique code to each case. Data collected were used strictly for the purposes of this investigation and not for any other purpose.

## Results

### Time–place–person description

**Fig. 1:**
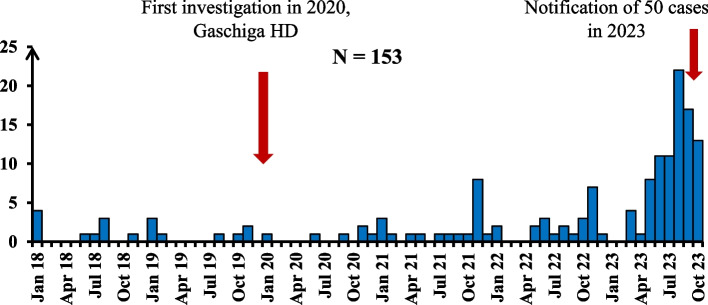
Epidemic curve of chronic ulcer cases after investigation in the study area, 2023

Before investigation, 50 skin ulcer cases were reported with onset from January 2018 to October 2023. During the in-depth investigation, we found 103 additional cases (total = 153 cases). The majority of the cases were recorded between May and October 2023 (Fig. 1).

**Fig. 2:**
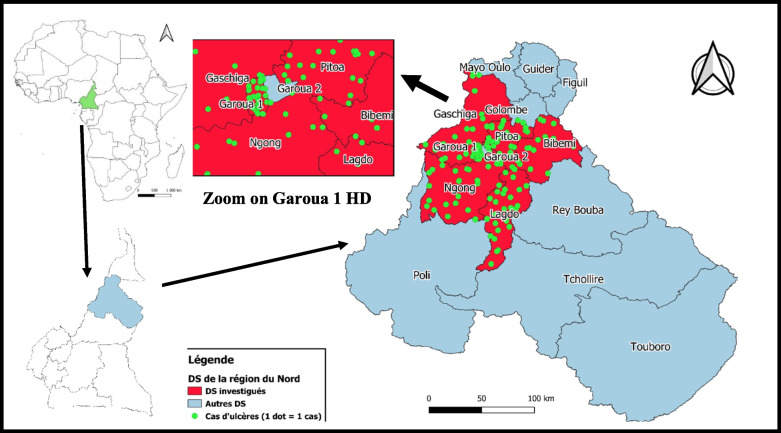
Geographical distribution of cases by HD, Northern Region, Cameroon, 2023

The distribution of cases in the study area in order of burden was: Garoua 1 (*n* = 32, 20.9%), Pitoa (*n* = 29, 19.0%), Lagdo (*n* = 28, 18.3%), Ngong (27, 17.6%), Gaschiga (*n* = 19, 12.4%), and Bibemi (*n* = 18, 11.8%) (Fig. 2). No cases was reported in Garoua 2 HD as seen in the zoomed-in region of the map.

The chronic ulcers found in the study area were derived from 18 HAs (Annexes 1–4, supplementary file): Bangli (*n* = 24, 15.7%), Bame (*n* = 15, 9.8%), Ndjola (*n* = 12, 7.8%), Pitoa (*n* = 12, 7.8%) and Langui (*n* = 11, 7.2%) were the main HAs affected with more than 10 cases. The other HAs were: Ngong (*n* = 9, 5.9%), Gaschiga (*n* = 8, 5.2%), Bascheo (*n* = 8, 5.2%), Djaloumi (*n* = 8, 5.2%), Djipporde (*n* = 7, 4.6%), Boumedjere (*n* = 6, 3.9%), Boula Ibib (*n* = 6, 3.9%), Bakona (*n* = 5, 3.3%), Padarme (*n* = 5, 3.3%), Bibemi (*n* = 5, 3.3%), Pomla Manga (*n* = 3, 2.0%) and Tongo (*n* = 1, 0.7%).

**Fig. 3:**
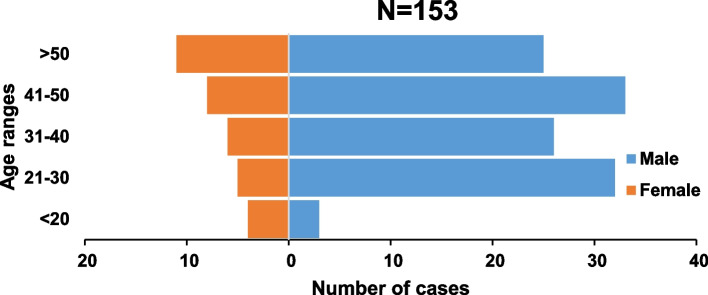
Distribution of ulcer cases by age group and sex in the study area, 2023

The distribution of cases in the study area by sex showed that men were most affected, with 119 (77.8%) cases, for a male/female sex ratio of 7:2. The median age of the cases was 38.5 years (9 months to 94 years). The most affected age group was 41–50 years (26.8%) (Fig. 3).

**Fig. 4:**
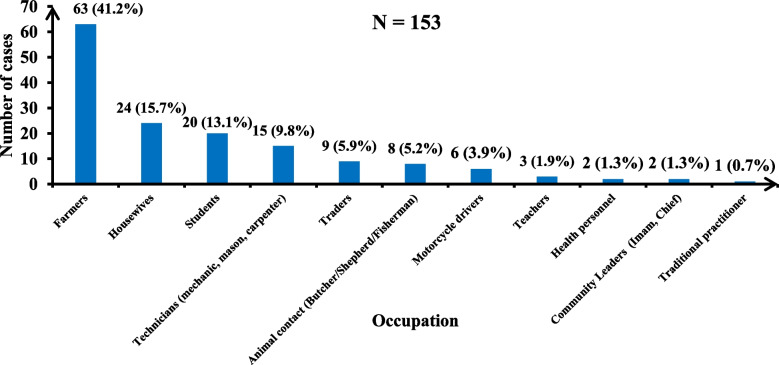
Distribution of ulcer cases by occupation in the study area, 2023

In terms of occupation, farmers (*n* = 63, 41.2%), housewives (*n* = 24, 15.7%), and pupils/students (*n* = 20, 13.1%) were the three most represented. In addition, two (1.3%) health workers and one (0.7%) traditional practitioner were affected (Fig. 4).

Other socio-demographic characteristics are listed in Table 1. Some are featured as follows: 95 (62.1%) patients were married, 57 (37.3%) did not go to school, 67 (43.8%) of the households were composed of 6 to 10 members, 118 (77.1%) patients lived in rural environment and 5 (3.3%) patients were known hypertensive (Table 1 below).

**Table 1:** Sociodemographic characteristics of cases in the study area, 2023

Variables	Number of cases	Percentage (%)
Marital status		
Married	95	62.1
Single	50	32.7
Divorced	1	0.7
Widow	7	4.6
Gender		
Male	119	77.8
Female	34	22.2
Level of education		
None	57	37.3
Primary	56	36.4
Secondary	37	24.2
Superior	3	2.0
Household number of people		
1 to 5	42	27.5
6 to 10	67	43.8
More than 10 (11–32)	44	28.8
Living environment		
Rural	118	77.1
Urban	35	22.9
Ethnic group		
Moundang	25	16.3
Guiziga	20	13.1
Guidar	18	11.8
Toupouri	12	7.8
Laka	9	5.9
Mafa	8	5.2
Mambaye	7	4.6
Others (Peul, Bata, Baiwawa, Massa, Fali, Gambai, Bororo, Daba, Foulbe)	36	23.5
Medical history		
Hypertension	5	3.3
HIV	1	0.7
Alcoholism	3	2.0
Smoking	2	1.3
Diabetes	0	0
Body Mass Index (BMI)		
Low (< 18,5)	1	0.7
Normal (18,5–24,9)	9	5.9
High (> 25)	4	2.7

**Fig. 5:**
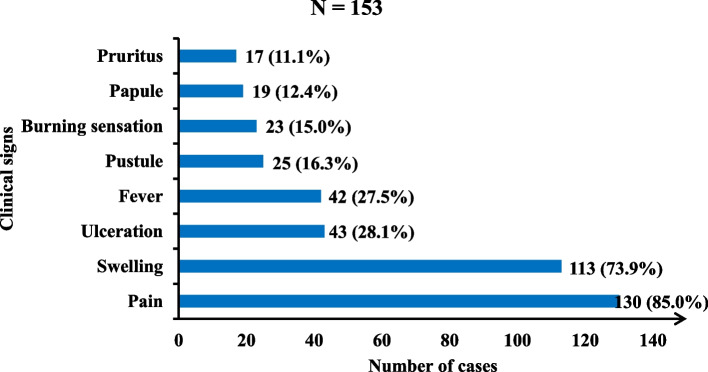
Clinical signs and symptoms at disease onset in the study area, 2023

Pain (*n* = 130, 85%) cases, swelling (*n* = 113, 73.9%), ulceration (*n* = 43, 28.1%), and fever in (*n* = 42, 27.5%) cases were the most frequent symptoms and signs (Fig. 5).

In 67 (43.8%) cases, the onset of ulceration was preceded by a trauma that created an entry point. Insect bites were mentioned in 7 (4.6%) cases. In more than half of the cases, i.e., 79 (51.6%) cases, no cause of the lesion was reported.

**Fig. 6:**
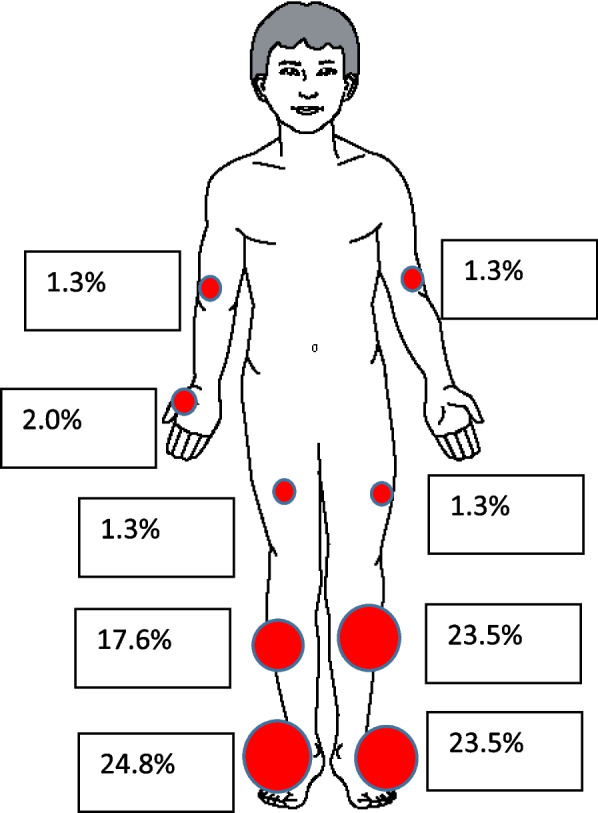
Location of ulcers on the body in the study area, 2023

In terms of location, 143 (90.2%) cases affected the lower limbs, and 10 (6.5%) affected the upper limbs. More specifically, the right foot (*n* = 38, 24.8%), left foot (*n* = 36, 23.5%), left leg (*n* = 36, 23.5%) and right leg (*n* = 27, 17.6%) were the four most frequent locations (Fig. 6).

**Fig. 7:**
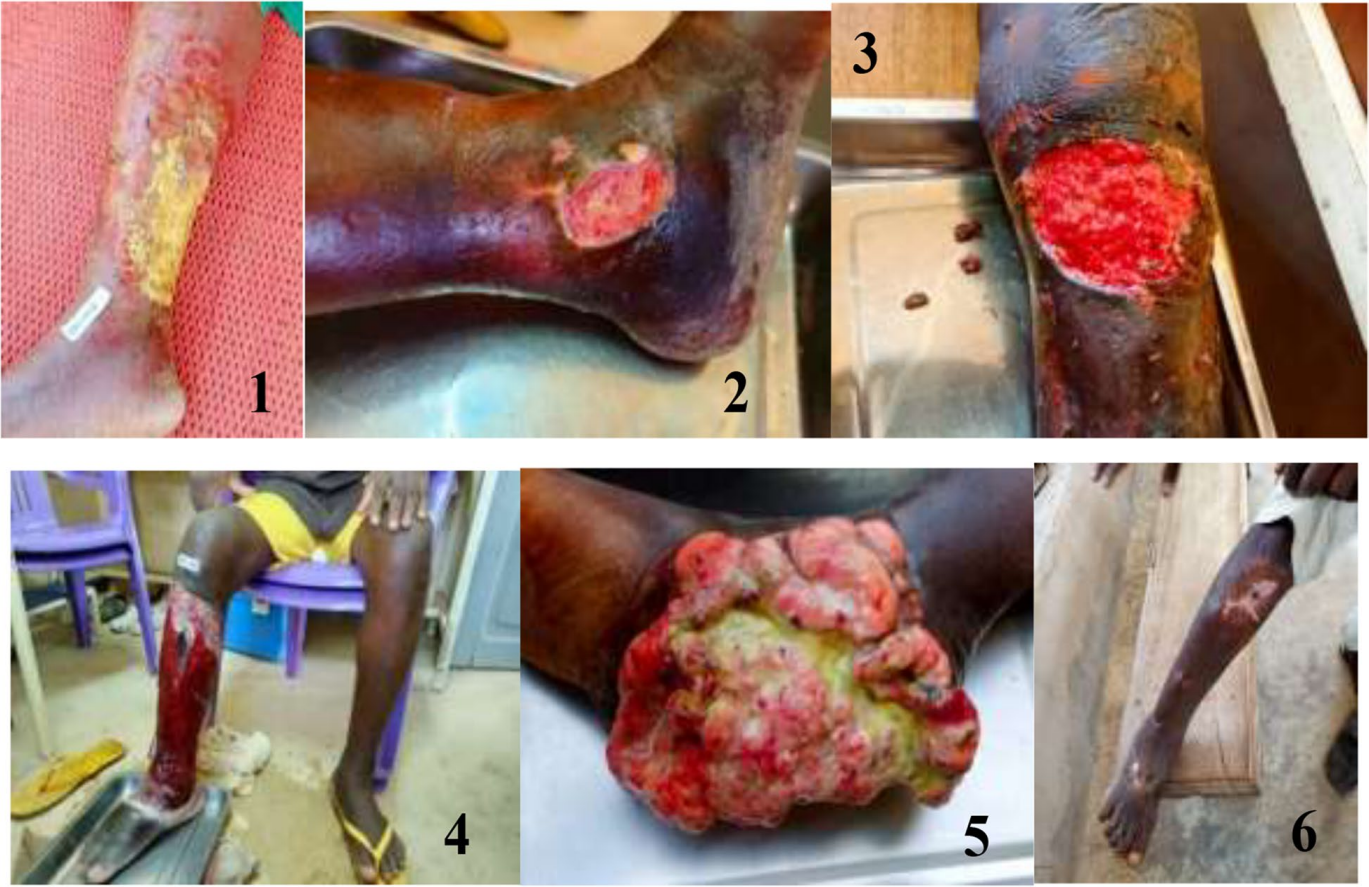
Images 1–6: Ulcers and lesions on different stages in the study area, 2023 (*From left to right).* 1-Erysipelas on the right leg. 2-Right ankle ulcer. 3-Left knee ulcer. 4-Large ulcer with right tibia necrosis. 5-Suspicion of cancer lesion, right ankle. 6-Healed ulcer, right leg

Evolving ulcers were the most common clinical form, accounting for 140 (89.7%) cases associated with lymphedema in 2 (1.3%) cases. Ten (6.4%) ulcer were healed at the time of investigation. One patient with suspected squamous cell carcinoma (skin cancer) was identified and biopsied (Fig. 7).

### Clinical description of lesions

Ulcers were mostly well-marked (*n* = 102, 66.7%) with regular edges (*n* = 92, 60.1%), sometimes deep (*n* = 67, 43.8%), with purulent discharge (*n* = 51, 33.3%), or hyperesthesia (*n* = 104, 68.0%), perilesional edema (*n* = 89, 58.2%), no associated adenopathy (*n* = 142, 92.8%) and preserved limb sensitivity (*n* = 145, 94.8%) (Table 2).

**Table 2:** Clinical description of ulcers in the study area, 2023

Items	Number	Ranges/Percentage
Number of ulcers	215 (Median: 1)	Min: 1; Max: 10
Ulcer size		
Length (major axis)	Average: 13 cm	1–41 cm
Width (minor axis)	Average: 7 cm	1–31 cm
Ulcer boundaries		
Well-marked	102	66.7
Poorly defined (crumbled)	36	23.5
Borders		
Regular	92	60.1
Irregular	47	30.7
Ulcer depth		
Superficial	69	45.1
Deep (Full skin or muscle/bone affected)	67	43.8
Ulcer bottom		
Clean	84	54.9
Purulent	42	27.5
Necrotic	12	7.8
Discharge		
Purulent	51	33.3
Blood	4	2.6
Clear discharge	12	7.8
No discharge	72	47.1
Ulcer sensitivity		
Hyperesthesia	104	68.0
Hypoesthesia	33	21.6
Healthy surrounding skin	79	51.6
Black skin border	58	37.9
Scaling of the soles of the feet	16	10.5
No desquamation	102	66.7
Clinical aspects associated with ulcers		
Old ulcer scars	19	12.4
Perilesional oedema	89	58.2
Lymph adenopathy	11	7.2
Limb sensitivity conserved	145	94.8
Distal pulses present	144	94.1
Toe space affected (mycosis, intertrigo)	6	3.9

### Treatment

**Fig. 8:**
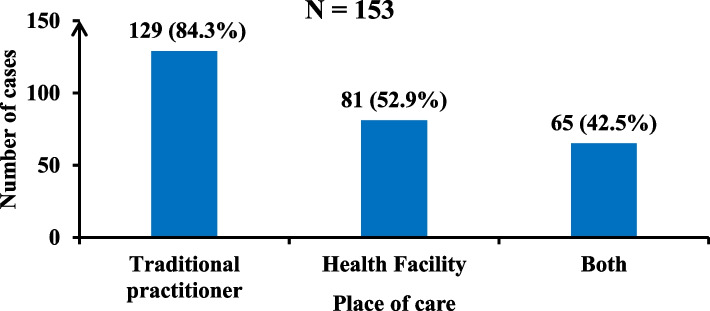
Place of care for cases in the study area, 2023

Among the 153 cases, 129 (84.3%) visited a traditional healer, 81 (52.9%) visited a health facility, and 65 (42.5%) visited both a health facility and a traditional healer (Fig. 8).

The treatment administered by the traditional healer was herbal tea using local plants (*n* = 98, 64.1%), poultices using a reddish powder (*n* = 95, 62.1%) produced from *Boswellia dalzielii* (Fufulde local name: Andakehi plant), dressings (*n* = 46, 30.1%), and shielding (*n* = 6, 3.9%). The treatment most frequently administered in health facilities included antibiotic penicillin (cloxacillin) in 78 (51.1%) cases, non-steroidal anti-inflammatory drugs (diclofenac) in 70 (45.8%) cases and dressings in 57 (37.3%) cases. These data are not presented in a figure or table. Ulcer cases found in the community were managed on site or in health facilities according to a defined protocol: Vaseline bandage, antalgics (Paracetamol, Tramadol), antibiotics (Amoxicillin or Amoxicillin-Clavulanic acid combination). A briefing was also carried out in the health facilities to ensure the implementation of the case management protocol.

### Results of biological analyses

After confirmation of the signed consent, we collected blood samples from 94 cases, three ulcer swabs per patient for 65 cases, and skin biopsies from 28 cases (Table 3). Cases of refusal were examined and managed in accordance with the established protocol.

**Table 3:** Types of samples collected by HD in the study area, 2023

Type of samples	Health Districts						Total
	**Pitoa**	**Ngong**	**Bibemi**	**Gashiga**	**Garoua 1**	**Lagdo**	**6**
**Whole blood**	9	13	13	16	35	8	94
**Ulcer swabs**	6	12	9	12	21	5	65
**Skin biopsy**	3	9	8	5	1	2	28
Total	**18**	**34**	**30**	**33**	**57**	**15**	**187**

The serological test results were as follows: six HIV-positive cases, seven TPHA-positive cases, and 1 TPHA + and VDRL + patient. No cases were double positive for HIV or TPHA. All positive cases were referred to the health facility for treatment in accordance with the national protocols. With respect to molecular analysis, PCR testing was negative for *Mycobacterium ulcerans* (Buruli ulcer) and *Haemophilus ducreyi* in all samples. Anatomopathology of the biopsies revealed necrotizing dermohypodermatitis (14/28) and botryomycosis or pyogenic granuloma (12/28) resulting from superinfection (extensive capillary vascular proliferation). One case of a smooth histiocytic tumor (clinically suspected to be squamous cell carcinoma) and one case of an inflammatory tumor were also found. Anatomopathological analysis revealed no leishmaniasis or leprosy (Table 4).

**Table 4:** Results of biological analysis in the study area, 2023

Analyses	Number of samples	Positive results	Negative results	Comments
TPHA serology	94	7	87	
VDRL serology	94	1	93	TPHA +/VDRL +
HIV Serology	94	6	88	
PCR Treponematosis (Syphilis, Yaws)	65	00	65	All Negative
PCR *Haemophilus ducreyi*	65	00	65	All Negative
PCR Buruli ulcer	65	00	65	All Negative
Anatomopathology of biopsies	28	-Necrotizing dermo-hypodermatitis (14) -Botryomycosis or pyogenic granuloma (12) -Smooth histiocytic tumor (1) -Inflammatory tumor (1) -Negative for leprosy and leishmaniasis		

### Entomological findings

Mosquitoes identified belonged to two genera (Culex and Anopheles) and six species. After biconical traps were used, two fly species were identified: *Stomoxys calcitrans* and *Tabanus tabanus* (Table 5). Phlebotomine vectors of leishmaniasis were not identified.

**Table 5:** Mosquito species obtained after entomologic investigation via biconical traps in the study area, 2023

Species	Health Districts					
	**Pitoa**	**Ngong**	**Bibemi**	**Gashiga**	**Garoua 1**	**Lagdo**
***Stomoxis calcitrans***	6	10	5	0	0	0
***Tabanus tabanus***	10	2	0	0	0	0
Total	16	12	5	0	0	0

### Results of the socio-anthropological survey

In all, we carried out 15 individual interviews and three focus group discussions. The groups were composed of community members: one group of men (21 years or more), one group of women (21 years or more), and one group of young people over 18. The results are presented in five themes identified from the participants: 1) social beliefs on Ladde disease; 2) manifestations of Ladde; 3) causes of Ladde; 4) treatment of Ladde; and 5) prevention of Ladde.

### Theme 1: Social beliefs of Ladde disease

The respondents labelled the concept of “Nyao Ladde” as linguistic borrowing whose origins lie in the Fufuldé language. Translated literally, it is understood in French as “Maladie de la brousse” and “Hendu le vent”. For most respondents, Ladde is a mystical pathology with diabolical origins. It is said to be caused by some kind of evil spirit. Selected responses are below.

“Ladde is a wind, a spirit that invades someone. It is invisible. No one can see it.”

“Ladde is not contagious. If it were, we'd all be sick by now. It is visible even in animals. It does not attack the whole herd”.

### Theme 2: Manifestations of Ladde

For most respondents, Ladde presents itself through a number of symptoms: fever, a burning sensation, itching, redness, and a purulent ulcer. Selected responses are below.

“At first, you feel a burning sensation; it is as if the fire has burned you, it heats up. Then, it starts to itch, and the more you scratch, the more the wound becomes infected. After a few days, if nothing is done, the ulcer starts to grow, and the flesh falls off.”

“It affects all parts of the body. For some, it is the feet; for others, it is the leg, the arm, the thigh; it affects the whole body”.

### Theme 3: The causes of Ladde, “the devil's disease”.

A reading of the participants'responses reveals that the causes of Ladde syndrome are not objectively known. Selected responses are below.

“The ladde is a diabolical spirit. He is found in trees. If, by misfortune, a twig from this tree bites you, you will directly catch this disease.”

“This disease has several causes. Some cases developed the disease after a cut with an object; others after a traffic accident; after a metal wound; a hoe crank, a millet stalk, an insect bite.”

“Medically speaking, we do not know what causes this pathology. We think of them as wounds, except that they're infected. Classic wounds are not as extensive.”

### Theme 4: Treatment of Ladde

Ladde’s disease is a pathology whose treatment remains unknown with respect to conventional medicine. For this reason, cases and their families are forbidden from visiting the hospital, as an injection would catalyze the spread of the disease and thus impair the patient's vital process. Selected responses are below.

“When we know that is what it is, we first go to the healer. It is true that we also go to the hospital for injections or bandages. However, here, we go to the Traditional healer first.”

“The Bororo traditional healers treat this. It is their disease. They're the ones who brought the disease to town. They have a powder that they apply to the wound, and it heals quickly.”

“People do not go to the hospital because of fear. They believe that as soon as they are injected, they will die. They even prefer to hide in their homes and only visit the hospital when the disease is already quite advanced. Others are even ashamed to go out with this disease; they trust the marabouts more”.

#### Collaboration between traditional healers and conventional medicine

In regard to treating Ladde, traditional healers claim that there is no real direct collaboration with hospitals. However, they recommend that their cases visit hospitals for dressings:

“I always advise cases to go for treatment, dressings. However, they do not want to. They prefer to come to me, but I always advise the hospital when I finish my incantations.”

“As soon as we have a case of Ladde, we report it to the hospital. We call them immediately, except that the population is convinced that this disease cannot be treated in the hospital. They think that if they go to the hospital, they will die. As soon as someone has Ladde, everyone bans them from the hospital. They often prefer to hide and come and see us when it is already advanced.”

### Theme 5: Preventing Ladde

It is clear from these comments that in local culture, there is no formal or effective means of combating Ladde. Selected responses are below.

“It is impossible to prevent Ladde. It is diabolical in origin. The ancestors could not prevent it; we found it that way. We cannot diagnose it, but we cannot prevent it.

“I do not truly know if this disease can be prevented. However, Father Bororo told us that it can be prevented by shielding. We armour you against evil spirits.”

## Discussion

We have provided a comprehensive investigation of chronic ulcers of unknown cause affecting Northern Cameroon. The main findings among the samples analysed were that of dermohypodermatitis followed by botryomycosis also called pyogenic granuloma, an inflammatory tumor of the skin and mucous membranes often caused by superinfection of minor traumatism [17]. A high percentage of them are preceded by a trauma that generates an entrance point. Recourse to hospital care is absent or infrequent, to the detriment of traditional practitioner care (84.3%). We also found that most cases resided in rural areas, an environment not always well covered in terms of quality care, a factor that could also favour superinfection of the initial wounds that was neglected or poorly managed. In view of the social representation of the ulcer as a spirit, we noted that in 43% of the cases, the onset was of traumatic origin, and in 51.6% of the cases, the spontaneous onset or exact mechanism was unknown, although the most dominant warning sign was pain. This result contradicts the findings of the anthropological study, which suggested that the Ladde was a diabolical spirit in the tree and that the lesions developed secondarily after contact with the tree. Clearly, awareness-raising is essential, and the referral of cases to a hospital that is qualified to identify and treat them in the initial phase is crucial to reversing this trend. Our study also revealed that our cases in households with more than five people, most of whom were the heads of their families or even spouses and children. This result could indicate a high level of skin contact in families with open wounds or the shared use of equipment used for wound care, on the one hand, and the recourse to traditional care over modern care due to the lack of financial means.

During this investigation, we did not obtain any samples of sandflies or *Tabanides chrysops* (horseflies). The environmental and climatic conditions may explain this, in that sandflies and tabanids are species that multiply more during the rainy season between July and the end of September in the Northern region [18]. Further research into cases of similar ulcers in domestic animals or livestock could provide more information as livestock can be infected with similar pathogens humans have and cross-infection can occur.

The results of a scientific study carried out as part of a doctoral thesis in history at the University of Ngaoundere listed the various factors involved in the spread of neglected tropical diseases (NTD) in the Far North region of Cameroon [19]. The natural propagation factors are climate and geography. Anthropogenic factors associated with the perpetuation of NTDs include the sociocultural habits of populations and economic factors linked to disease endemicities [19].

Differential diagnosis of chronic ulcers in the Northern region include leprosy, yaws and Buruli ulcer. A study carried out in 2010 in the Mape basin in the southwestern Adamaoua region revealed 32 cases of leprosy, 29 cases of yaws and 25 cases of Buruli ulcer, on the basis of clinical symptoms [20]. With 32 cases of leprosy, the population-adjusted prevalence was 6.5 cases per 10,000. The majority (70%) of identified leprosy cases suffer from the multibacillary form of the disease [20]. Analyses of the biological samples returned to favour the multibacillary form [20]. These diseases (leprosy, yaws, and Buruli ulcer) are characterized by chronic skin and soft tissue lesions, which predispose affected individuals to secondary bacterial infections, including bacterial dermohypodermatitis. Specifically, the multibacillary form of leprosy can cause extensive ulcerations and nerve damage, Buruli ulcer results in deep, necrotic skin lesions.

Analyses of biological samples supported the major clinical diagnosis of necrotizing dermo-hypodermatitis without identifying a precise causal germ. None of the cases were PCR positive for *Mycobacterium ulcerans*, the causative agent of Buruli ulcer. In 2020, laboratory analysis (pus examination) isolated *Streptococcus pyogenes, Staphylococcus aureus, Klebsiella pneumoniae,* and *Escherichia coli* in the samples collected in Gashiga HD [13]. Blood culture results were all negative [13]. Buruli ulcer was clinically suspected but *Mycobacterium ulcerans* was not isolated [13].

Additional bacteriological tests could have been carried out this time to search for bacteria common to the skin microbiome, but infectious once the skin barrier is breached, such as Group A *Streptococcus* [21], *Staphylococcus aureus* [21] or *Pseudomonas aeruginosa* [22, 23]. These bacteria have varying levels of antimicrobial resistance, which can complicate laboratory testing, confirmation, and treatment options [24–26]. In yaws-endemic areas, two-thirds of exudative cutaneous ulcers are associated with *Treponema pallidum subsp. pertenue* and *Haemophilus ducreyi*; one-third are classified as idiopathic ulcers [27]. Also, the aetiology of idiopathic ulcers in tropical climates is still not well understood but the pattern that emerges using more advanced diagnostic techniques suggests a complex picture where there are many potential bacterial causes some of which act in synergy. The results of a study confirm the association of *S. pyogenes* with idiopathic ulcers in yaws-endemic areas, and suggest that additional anaerobic bacteria, but not other microorganisms, may be associated with this syndrome [27].

Management of cases of bacterial dermohypodermatitis includes early antibiotic therapy with penicillin M (amoxicillin effective against *Streptococcus* or amoxicillin-clavulanic acid effective against beta-lactamase-secreting gram-negative bacteria at 3 g/day for adults for 10–14 days) [28], that are reasonable empirical choices however they should be adjusted based on the culture results and antimicrobial susceptibility testing and after seeking specialist advice if required. Other measures include pure analgesics (paracetamol, tramadol), NSAIDs are contraindicated, regular Vaseline dressings (according to distributed protocols), biological check-up for comorbidities (fasting blood sugar, HIV serology), and monitoring and control of underlying diseases (hypertension, diabetes, obesity). Case counselling on treatment compliance and hospital management is also necessary [28].

### Limitations

Our study has some limitations. The lack of equipment for measuring parameters on all cases (bathroom scales, blood pressure monitor), the failure to perform fasting blood glucose tests, and the descriptive nature of the study were the major limitations. A case–control study could be carried out to identify risk factors. Further studies possible would include consideration of common skin flora that are infectious once the skin barrier is broken, viral and fungal pathogens, incorporate reviews of zoonotic (wildlife and domestic animal) skin disease data. Application of more involved statistical analysis methods (e.g., backward stepwise regression) to these study data may identify combinations of patient characteristics, exposures, and risks. Further studies could also address awareness of similar skin infections in livestock (e.g., cattle, goats, dogs).

## Conclusion

In summary, 215 chronic ulcers were recorded in 153 cases between January 2018 and October 2023 in the study area. Socio-demographically, men were four times more affected than women, with a median age of 38.5 years. Leg ulcers were the most common clinical location. The notion of trauma creating an entry point was found in more than 40% of cases, and insect bites were found in 4% of cases. More than eight out of 10 patients sought care from a traditional practitioner, and one out of two patients visited a health facility. The results of the bacteriological and anatomopathological analyses revealed necrotizing dermohypodermatitis. At the end of the study, we recommended that healthcare staff carry out screening tests for diabetes (fasting glycemia) and HIV in all cases with chronic ulcers. Supplying health facilities with case management inputs, building the capacity of health and laboratory staff in the diagnostics and management of chronic ulcers (including Buruli ulcer), and setting up a case management unit (skin grafts) at Garoua Regional Hospital would be appropriate measures to improve the prognosis of cases. In addition, improving community engagement in healthcare and good collaboration between health personnel and traditional practitioners would be useful for better case follow-up.

### Patient follow-up

Four cases referred to the traumatologist received skin grafts. A patient with the smooth histiocytic tumor died on 20th January 2024.

## Supplementary Information

Supplementary Material 1.

## Data Availability

Data is provided within the manuscript and the databases used and/or analysed during the current study are available from the corresponding author on reasonable request.
