# Supplementary material for: Investigation of chronic limb ulcers in Northern Cameroon: a socio-anthropological and clinical perspective
Source: BMC Infect Dis. 2025 Jul 1;25:842. doi: 10.1186/s12879-025-11251-4 (PMC12211150; doi:10.1186/s12879-025-11251-4)
Supplement: Supplementary file 1 — Supplementary Material 1. [file 12879_2025_11251_MOESM1_ESM.pdf]

<u>Interview guide for health personnel</u>

Always provide reminders for each section

Respondent Identification	• Gender - Age<br>• Occupation - Position<br>• Department - Number of years of service<br>• District/location
Description of chronic ulcer cases in Time place and person in the North Region	• Knowledge: In your opinion, what is "Ladde"?<br>• Manifestation: How does it manifest itself?<br>• Symptom: What was the sign/first symptom you observed?<br>• Affected body zones: Where are they located on the body?
Identify the cause and source of chronic "Ladde" ulcers	• Causes: What factors do you think may be responsible for "Ladde"? In your opinion, is this disease linked to water? mosquitoes? mysticism?<br>• Manifestations: What are the causes and manifestations of "Ladde"?
Social representations of "Ladde" by the community and health personnel	• What are your perceptions of Ladde?<br>• What are people's perceptions of people with Ladde?<br>• Are there any categories of people who have a specific perception of Ladde? If so, which ones?<br>• In your opinion, which groups of people are most at risk of becoming seriously ill if they contract Ladde?<br>• How do people in the community regard those suffering from "Ladde"? How are they treated?
Determining the therapeutic itinerary of cases	• Consultation: Do "Ladde" patients come for consultation at the health facility? If so, how often?<br>• Offer: Do you have an effective treatment to offer "Ladde" patients?<br>• Collaboration with: Do you collaborate with traditional healers to treat patients?<br>• If so, how?
Education and communication	• What awareness-raising activities do you carry out concerning Ladde?<br>• How do patients and the general public react to awareness-raising messages about Ladde?
Difficulties and suggestions	• What particular difficulties do you encounter in preventing and managing Ladde?<br>• How do you overcome these difficulties?<br>• What suggestions do you have for improving the prevention and management of "Ladde"?

Interview guide for community leader

Respondent identification	• Gender<br>• Age<br>• Occupation<br>• Role in the community as a leader<br>• - District/location
Description of cases of chronic ulcers in Time place and person in the North Region	• In your opinion, what is 'Ladde'?<br>• How does it manifest itself?<br>• What was the first sign/symptom you observed?<br>• Where does it affect the body?
Identify the cause and source of chronic Ladde ulcers	• What factors do you think may be responsible for Ladde?<br>• What are the causes and manifestations of Ladde?<br>• In your opinion, is this disease linked to water? mosquitoes? mystics?
Social representations of Ladde in the community	• What are your perceptions of Ladde?<br>• What are people's perceptions of people with Ladde?<br>• Are there categories of people who have a specific perception of Ladde? If so, which ones?<br>• In your opinion, which groups of people are most at risk of becoming seriously ill if they contract Ladde?
Determining the therapeutic itinerary of cases	• Where do you think Ladde is treated? (Hospital or pharmacopoeia? Follow-up)<br>• What do you think patients in your community do when they suffer from Ladde?<br>• What role do traditional healers play in the management of this disease? Tell us about it
Role in the fight against Ladde	As a leader, how are you involved in the fight against Ladde?
Difficulties and suggestions	• What difficulties does your community face in preventing and treating Ladde?<br>• What suggestions do you have for improving the fight against this disease?

<u>Interview guide for traditional practitioners</u>

Theme 1: Introduction and presentation

. Tell us about your work as a traditional healer

What are the taboos (food, cultural activities, etc.) towards patients?

Theme 2: Knowledge of the causes and manifestations of Ladde

- Do you know about Ladde?
- What are the causes?
- How does it manifest itself?
- What do you think illness or health is?
- What do you call this illness in your language? (What does Ladde mean in your culture? Social representations of the disease)

Theme 3: LADDE treatment protocol

- How can you tell the difference between Ladde and other similar diseases?
- How do you treat this disease?
- What do you tell them to do or not to do to combat this disease?
- Why do patients come to you?
- How effective is your treatment?

Theme 4: social representations of the disease

- How would you describe people suffering from Ladde and their families?
- What should be done to prevent Ladde?
- What do people say about Ladde? What do they think about this disease?
- . How do people in the community regard those suffering from Ladde? How are they treated?

Theme 5: Collaboration with modern health services, difficulties and suggestions

- How do you collaborate with health staff and the hospital in caring for patients?
- What difficulties do you face?
- What proposals or complaints do you have?

Interview guide for Patients

Respondent identification

- Gender
- Age
- Occupation
- Position
- Department
- Number of years of service
- District/location

Description of cases of chronic ulcers in Time place and person in the Northern Region

- How did it start? How long has it been going on?
- What was the sign/first symptom you observed?
- Where are the ulcers located on the body?
- How is Ladde transmitted?
- Where are people with Ladde most likely to be found?

Identify the cause and source of chronic 'Ladde' ulcers

- For you, what are the causes and manifestations of 'Ladde'?
- What water sources do you frequent?
- Have you travelled recently?

Social representations of 'Ladde' by the community and health personnel

- For you, what is 'Ladde'?
- How dangerous do you think Ladde is?
- Do you think Ladde can be treated in hospital?

Determining the therapeutic itinerary of cases

- Since the onset of the disease, describe in chronological order the various therapeutic approaches you have used to date.
- Describe how you have enjoyed the treatment.
- How can Ladde be prevented?

Relationship between carer and patient (parents/family only)

- Why didn't you go directly or stay at the health centre?
- How were you received at the hospital?
- What impression do you have of the staff at the health centre?
- How do you find the health centre's services?

Difficulties and expectations

- What difficulties are you facing with this illness?
- What are your wishes and expectations?

Guide for Focus Group Discussion (FGD)

Identification of participants

- Gender
- Age
- Occupation
- District/location

Knowledge of Ladde

- In your opinion, what is ‘Ladde’?
- How does it manifest itself?
- What was the sign/first symptom you observed?
- What are the locations on the body?

Causes of Ladde

- In your opinion, what factors may be responsible for Ladde?
- In your opinion, is this disease linked to water? mosquitoes? mystics?

Social representations of Ladde

- What are your perceptions of Ladde?
- What are people's perceptions of people with Ladde?
- Are there categories of people who have a specific perception of Ladde?
- If so, which ones? In your opinion, which groups of people are most at risk of becoming seriously ill if they contract Ladde?

Therapeutic itinerary

- In your opinion, where is Ladde treated? Hospital or pharmacopoeia, recovery, what do patients in your community do when they suffer from ‘Ladde’?
- What role do traditional practitioners in the community play in treating this disease? Tell us about it?

Fight against Ladde

At your level, how are you involved in the fight against ‘Ladde’?

Difficulties and suggestions

- What difficulties do you encounter in the fight against Ladde?
- How can we help you in this fight?

Annexes

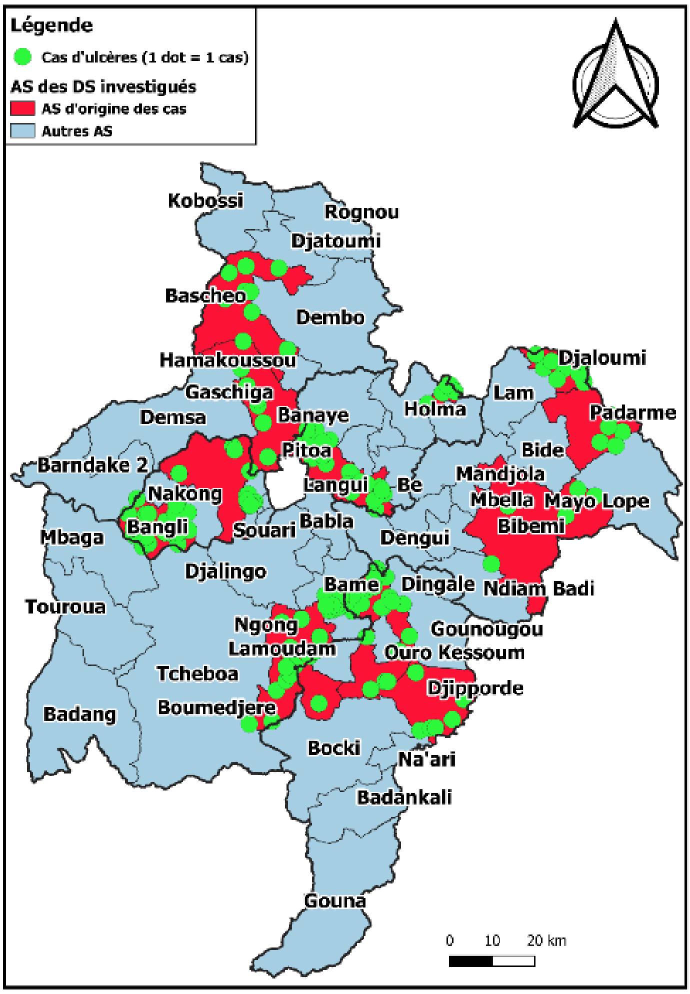

Annexe 1: Geographical distribution of cases by health areas in the study area, 2023

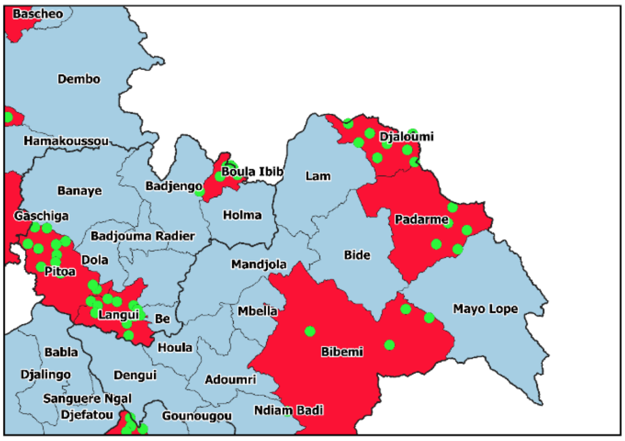

Annexe 2: Distribution of cases by HA in Bibemi and Pitoa HD, study area, 2023

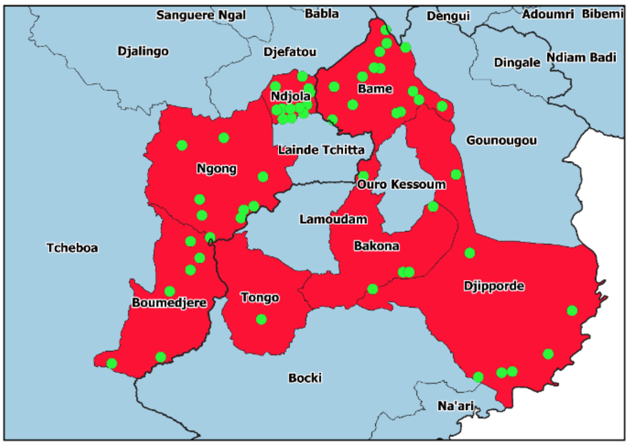

Annexe 3: Distribution of cases by HA in Lagdo and Ngong HD, study area, 2023

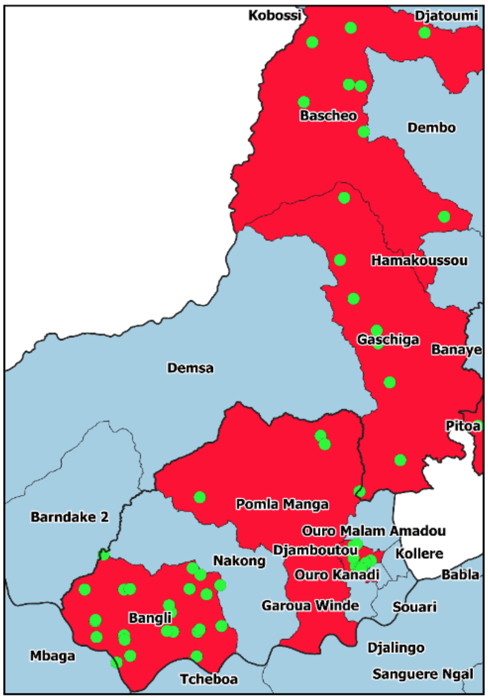

Annexe 4: Distribution of cases by HA in Garoua 1 and Gaschiga HD, study area, 2023
